# The Posterior Epidural Ligaments: A Cadaveric and Histological Investigation in the Lumbar Region

**DOI:** 10.5402/2013/424058

**Published:** 2013-10-02

**Authors:** M. J. Connor, S. Nawaz, V. Prasad, S. Mahir, R. Rattan, J. Bernard, P. J. Adds

**Affiliations:** ^1^Division of Biomedical Sciences (Anatomy), St. George's University of London, Cranmer Terrace, London SW17 0RE, UK; ^2^Department of Orthopaedics, St. George's Healthcare NHS Trust, London SW170QT, UK; ^3^Department of Histopathology, Frimley Park Hospital NHS Trust, London GU16 7UJ, UK

## Abstract

*Purpose*. Incidental durotomy is a relatively common complication for patients undergoing posterior spinal surgery. Delineating anatomical variants in the posterior lumbar spinal canal is crucial in reducing future rates of incidental durotomy. *Materials and Methods*. The ligamentous attachments between the dura mater and ligamentum flavum in the lumbar region of 17 soft-fixed cadavers were investigated. The lumbar vertebral columns were removed, and cross-sectional dissection was performed at levels L1-S1. Anterior retraction of the dorsal dura mater identified attachments between the dorsal surface of the dura mater and the ligamentum flavum. Histological staining of the ligamentous attachments was carried out with hematoxylin and eosin (H&E) and elastic van Gieson (EVG). *Results*. Posterior epidural ligaments were present in 9 (52.9%) cadavers. Nine (9) separate ligaments were identified in these cadavers, with 3 (33.3%) at L3/L4, 5 (55.5%) at L4/L5, and 1 (11.1%) at L5/S1. Histology confirmed the presence of poorly differentiated collagen-based connective tissue, distinct from the normal anatomy. *Conclusions*. This study confirms the presence of multiple dorsomedial posterior epidural ligaments at the main sites for posterior spinal surgery (L3-S1). An intraoperative awareness of the variability of such connections may be an important step in reducing static rates of incidental durotomy.

## 1. Introduction

Low back pain is a highly prevalent complaint, reported to affect 26.4% of US adults at some point in the last three months [1], although fewer than 1% of patients proceed to undergo surgical intervention [2]. In spite of methodical advances in surgical techniques, incidental durotomy (iatrogenic dural tears) during posterior spinal surgery still occurs.

Incidental durotomy or iatrogenic dural tears remain a relatively commonplace and potentially serious complication of lumbar spine surgery [3–5]. Delineating anatomical variants in the posterior lumbar spine may be crucial in reducing future rates of incidental durotomy. The aim of this study was to investigate variations in the anatomical and histological features of the posterior epidural ligaments, passing between the dura mater and the ligamentum flavum in the lumbar spine.

## 2. Materials and Methods

Seventeen lumbar spines were dissected from randomly selected soft-fixed cadavers with no known history of spinal disease or spinal surgery. Cross-sectional anatomical dissection at the levels L1-S1 via a dorsal approach was performed. Anterior retraction of the dura mater identified any connection between the dorsal surface of the dura mater and the ligamentum flavum. The anatomical features of those sites with macroscopic connections were noted and photographed.

Any attachments between the dura and the ligamentum flavum were dissected out in their entirety, fixed in 10% formalin and stained with hematoxylin and eosin (H&E) and elastic van Gieson (EVG). A number of randomly selected control specimens from levels L1-S1, without connection, were also stained with H&E and EVG. Staining with EVG differentiated collagen from elastin in the tissue structure excised.

## 3. Results

**Figure 1:**
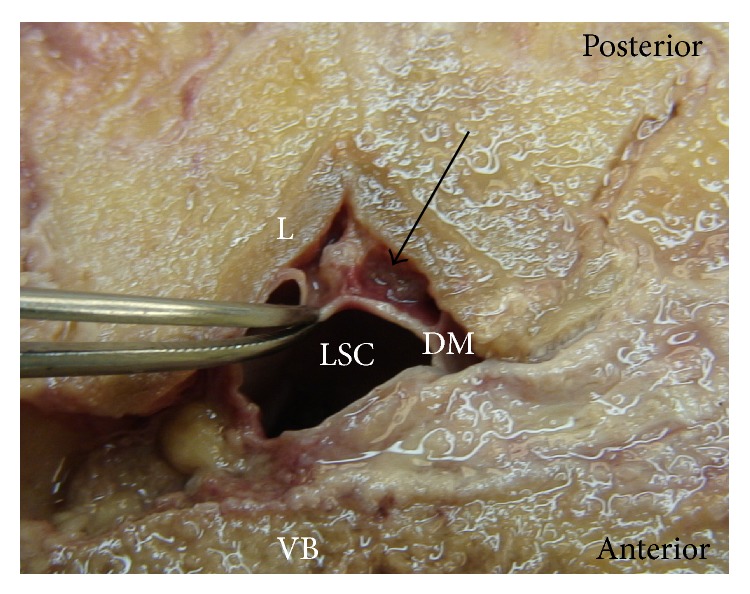
Photographic image shows a cross-sectional anatomical dissection of the lumbar spine at the L3/L4 vertebral level. Forceps illustrate anterior retraction of the dorsal surface of the dura mater to reveal the posterior epidural ligament (arrow). LSC: Lumbar spinal canal, VB: vertebral body, DM: dura mater, and L: lamina.

**Figure 2:**
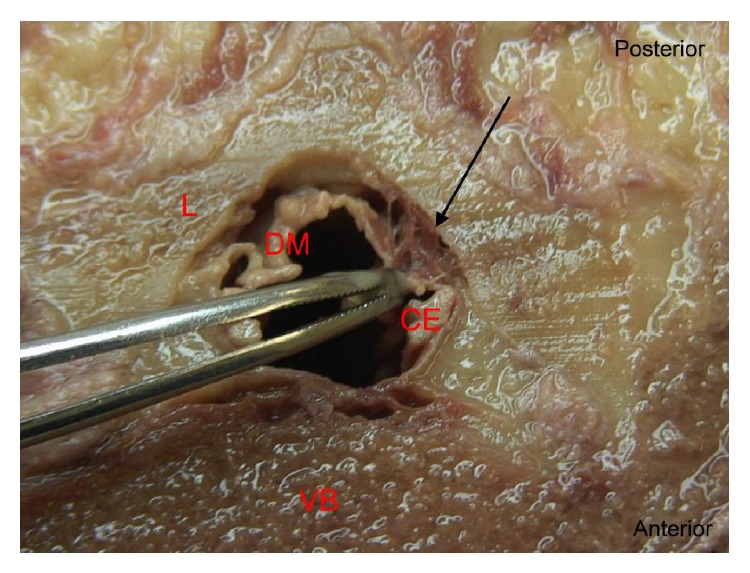
Photographic image shows a cross-sectional anatomical dissection of the lumbar spine at the L5/S1 vertebral level. Forceps illustrate anterior retraction of the dorsal surface of the dura mater to reveal the posterior epidural ligament (arrow). CE: cauda equine, VB: vertebral body, DM: dura mater, and L: lamina.

Posterior epidural ligaments were identified as a connection between the dorsal surface of the dura mater and the ligamentum flavum. These ligaments were present in 9 (52.9%) cadavers. Nine (9) separate posterior epidural ligaments were identified in these cadavers, with 3 (33.3%) at L3/L4, 5 (55.6%) at L4/L5, and 1 (11.1%) at L5/S1 (Table 1). Figures 1 and 2 demonstrate the variable presentations of the ligament and its identification at different vertebral levels.

**Figure 3:**
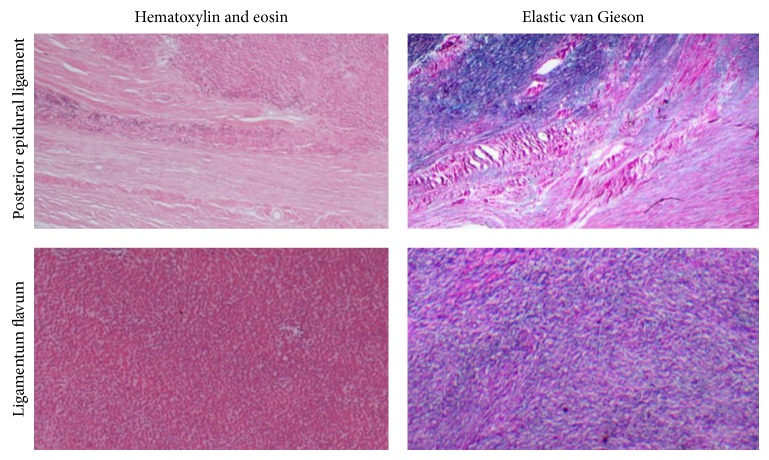
Comparative images of control ligamentum flavum and the identified posterior epidural ligament stained with H&E and EVG. Ligamentum flavum is 80% elastin and 20% collagen. Dura mater is a dense collagenous connective structure. On elastic van Gieson staining, elastin appears dark blue and collagen pink (H&E ×40, EVG ×40).

**Figure 4:**
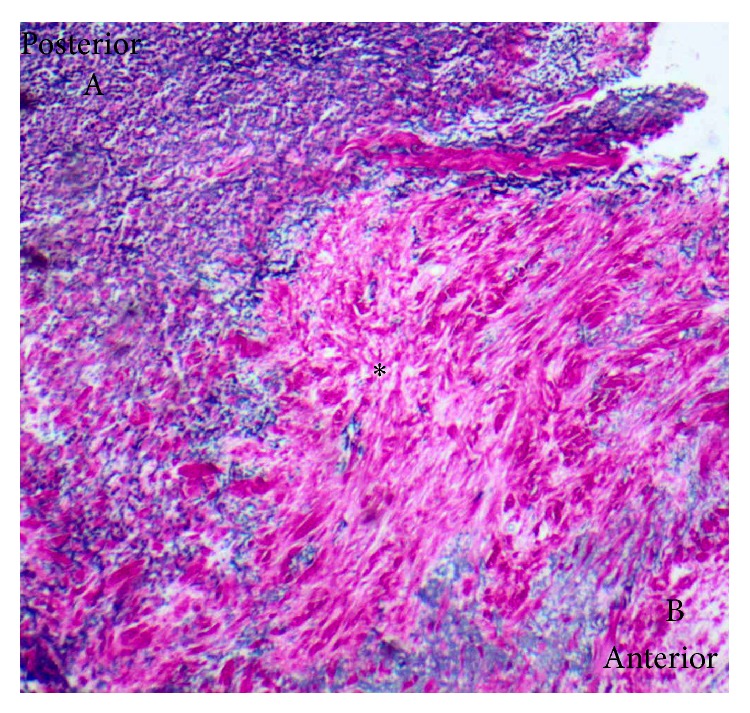
Histological slide showing the presentation of a posterior epidural ligament with a large coalition of tissue. Ligamentum flavum (A), dura mater (B), and posterior epidural ligament (∗) (EVG ×40).

**Figure 5:**
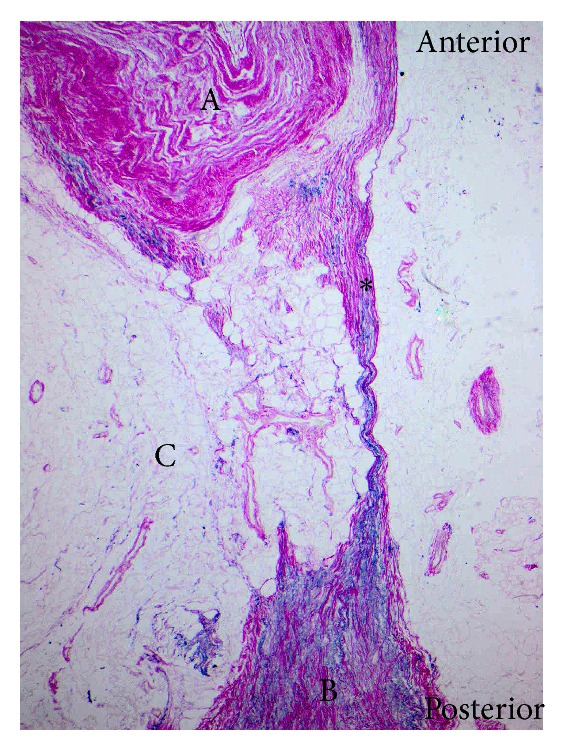
Histological slide showing a discrete stranded posterior epidural ligament compartmentalising the posterior epidural space. Dura mater (A), ligamentum flavum (B), posterior epidural fat pad (C), and posterior epidural ligament (∗) (EVG ×40).

Histology confirmed the presence of poorly differentiated collagen-based connective tissue, distinct from the normal anatomy (Figure 3). Histological analysis highlighted variants in the presentations of the ligament (Figures 4 and 5).  Figure 4 shows a ligament with a substantial coalition and fusion of fibres, which are distinct from the standard tissue plane. On other occasions, the ligament was observed as a discrete stranded structure with ventral-dorsal interdigitation of fibres (Figure 5).

## 4. Discussion

Despite methodical approaches and improved surgical technique, the incidence of incidental durotomy in posterior lumbar spine surgery has not significantly decreased. Unidentified incidental durotomy has a clear postoperative risk, and the existence of variants in the lumbar spine anatomy has been hypothesised as a possible cause for the stagnant incidence rates of incidental durotomy.

Previous anatomical studies have delineated ventral lumbar dural adhesions, which authors have reported them to be an adjuvant source of discogenic back pain [6]. In the posterior spine, lateral (Hoffman's ligaments) [7], dorsolateral, and dorsomedial epidural attachments have been described (reviewed by Kimmel et al. [8]). The “attention to terminal attachment” (ATA) has been described originating from the dorsal surface of the dura mater at the level of S1 projecting towards the ligamentum flavum [9]. This intraoperative finding had similar histological features to those identified in this anatomical study and was also described as a possible causative factor in dural tears. However, the dorsal ligaments described here are clearly different from the ATA as they were found at levels L3 to S1 and present a clear extension from the dorsal dura mater to the ligamentum flavum.

Posterior decompression of lumbar neural structures often involves removal or part removal of the lumbar lamina and ligamentum flavum. In patients with severe stenosis, surgeons regularly encounter adherent ligamentum flavum to the dura mater, where no epidural fat is present to define the surgical plane [10]. This study highlights the reasons why this may occur. We observed variable ligamentous attachments between the dorsal surface of the dura mater and ligamentum flavum at levels L3-S1. These ventral-dorsal ligaments tethered the dura mater to the ligamentum flavum forming a substantial communication (Figures 1 and 2). The presence of such ligaments, in the anatomical plane where lumbar spine surgery is most commonly undertaken, may have a role in the aetiology of intraoperative incidental durotomy.

Histology staining confirmed the presence of collagen-rich tissue that was distinct from the normal elastic structure of the ligamentum flavum (Figure 3). The variable histological presentations of the ligaments were outlined (Figures 4 and 5). The histological union of the dura mater and ligamentum flavum makes the intraoperative identification of separate tissue structures difficult, and care should be taken during surgery [11, 12].

With the knowledge of the existence of the posterior epidural ligament at levels L3-S1, the authors advocate surgical caution in the region with the employment of complication avoidance techniques when approaching the ligamentum flavum. The authors support the previous literature [10, 11] that recommends that two-step flavotomies, using semisharp dissectors, during a posterior approach to the lumbar spine should be standard surgical practice. Furthermore, with knowledge of the features of the posterior epidural ligaments, it is recommended that in cases where movement of the dura is visualized, areas of adherent ligamentum flavum should be left *in situ* [12].

## 5. Conclusion

The presence of ligamentous attachments between the dorsal surface of the dura mater and the ligamentum flavum is of surgical and anatomical importance. In this paper, we show these ligaments arising at vertebral levels L3-S1 in over half of the cadavers investigated and describe their varied anatomical and histological presentation. The delineation of the posterior epidural ligaments in the lumbar spine is of clear clinical importance in posterior lumbar spine surgery.

## Figures and Tables

**Table 1:** The posterior epidural ligaments identified during dissection and the corresponding vertebral levels.

Lumbar spine level^†^	Posterior epidural ligament (*n*)	Percentage total (%)
L3/L4	3	33.3
L4/L5	5	55.6
L5/S1	1	11.1

Total	9	100.0

^†^No ligaments were identified at L1-L2.

## References

[1] Deyo R. A., Mirza S. K., Martin B. I. (2006). Back pain prevalence and visit rates: estimates from U.S. national surveys, 2002. *Spine*.

[2] Weinstein J. N., Lurie J. D., Olson P. R., Bronner K. K., Fisher E. S. (2006). United States' trends and regional variations in lumbar spine surgery: 1992–2003. *Spine*.

[3] Cammisa F. P., Girardi F. P., Sangani P. K., Parvataneni H. K., Cadag S., Sandhu H. S. (2000). Incidental durotomy in spine surgery. *Spine*.

[4] Tafazal S. I., Sell P. J. (2005). Incidental durotomy in lumbar spine surgery: incidence and management. *European Spine Journal*.

[5] Khan M. H., Rihn J., Steele G. (2006). Postoperative management protocol for incidental dural tears during degenerative lumbar spine surgery: a review of 3,183 consecutive degenerative lumbar cases. *Spine*.

[6] Parke W. W., Watanabe R. (1990). Adhesions of the ventral lumbar dura. An adjunct source of discogenic pain?. *Spine*.

[7] Wadhwani S., Loughenbury P., Soames R. (2004). The anterior dural (Hofmann) ligaments. *Spine*.

[8] Kimmell K. T., Dayoub H., Shakir H., Sincoff E. H. (2011). Spinal dural attachments to the vertebral column: an anatomic report and review of the literature. *Surgical Neurology International*.

[9] Solaroglu I., Okutan O., Beskonakli E. (2011). The ATA and its surgical importance: a newly described ligament lying between the dural sac and the ligamentum flavum at the L5 level. *Spine*.

[10] An H. S., Jenis L. G. (2006). *Complications of Spine Surgery: Treatment and Prevention*.

[11] Herkowitz H. N., Dvorak J., Bell G. (2004). Section V: specific clinical entities. *The Lumbar Spine*.

[12] Kraemer R., Wild A., Haak H., Herdmann J., Krauspe R., Kraemer J. (2003). Classification and management of early complications in open lumbar microdiscectomy. *European Spine Journal*.

